# Mapping capacity building programs in health diplomacy: Relevance and application in an uncertain world

**DOI:** 10.12688/f1000research.134689.1

**Published:** 2023-07-13

**Authors:** Sanjay Pattanshetty, Aniruddha Inamdar, Kiran Bhatt, Viola Savy Dsouza, Anirudh Prem, Helmut Brand

**Affiliations:** 1 Centre for Health Diplomacy, Department of Global Health Governance , Prasanna School of Public Health (PSPH), Manipal Academy of Higher Education, Manipal, Karnataka, 576104, India; 2 Department of International Health, Care and Public Health Research Institute – CAPHRI, Faculty of Health Medicine and Life Sciences, Universiteit Maastricht, Maastricht, Limburg, The Netherlands; 3 Centre for Regulatory Science, Department of Health Information, Prasanna School of Public Health, Manipal, Karntaka, 576104, India; 4 Department of Health Policy, Prasanna School of Public Health, Manipal Academy of Higher Education, Manipal, Karnataka, India

**Keywords:** Health Diplomacy, Capacity Building Programs, Review, Global North, Global South, Global Health Diplomacy Programme

## Abstract

**Background:** Health diplomacy is one of the emerging avenues for academics where foreign policy dynamics and global health meet. Its relevance has augmented especially after the COVID-19 pandemic that brought the world to a halt. International organization and national entities that are responsible for health governance as well as its socio-economic determinants have been increasingly involved in the negotiations for a collective action towards a better health infrastructure and preparedness. However, the approach to health diplomacy seems to vary with whether health is looked through diplomacy lens or vice versa. Thus, inculcating adequate and appropriate competencies of both fields to conduct negotiations for health while keeping national interests and international commitments intact is imperative.

**Methods:** This study investigates 50 programmes/courses that have been currently offered around the globe to understand the competencies that have been identified as essential for a health diplomat. We examined four aspects: i) geographical distribution of programme/course (ii) the type of global health diplomacy programme being offered and their duration (iii) mode of teaching and (iv) cross-cutting themes that the programme offers.

**Results:** We found that the courses/programmes have been mostly provided by the countries of the Global North who play a key part in international negotiations. Although there were diverse types of certifications identified, they can be classified into two groups - core health diplomacy and inclusive health diplomacy programmes. The health diplomacy training is preferred to be provided in-person due to the nature of the work.

**Conclusions:** While competencies for health governance and international relation have been dominant among the current programmes, other cross-cutting themes such as economics, politics, law, public policy, crisis management, environment and public health have been considered essential. The article concludes with a proposal of a framework to streamline the sectors and the competencies that is required in health diplomats.

## Introduction

The contemporary world is experiencing uncertainty and adaptability challenges considering the disruptive development and globalization of diverse sectors. Globalization has made it evident that any given sector cannot function in silos. ^1^^–^^3^ Interdependence as a theory and practice is a necessity to be implemented in complex adaptive systems, which by nature are unpredictable. The health sector is no different in this context. ^4^ Emerging and re-emerging diseases have challenged the transnational health systems, services, infrastructure, leadership, and governance set by the institutions established after World War II. ^5^^,^^6^ The World Health Organization (WHO) was set up in 1948 as a progressive agency that set norms, standards, and guidelines to promote health and well-being, established partnerships through successful diplomatic and political negotiation with nations, partners, and people to ensure safety and protection of the vulnerable segments of the population. However, the growing discontent of globalization, political upheaval, food crisis, economic crisis, energy crisis, conflicts, cyber security, trade, intellectual property tensions, and technology wars have cornered the international organization to revisit their strategy in handling social, economic, political, and commercial determinants of health outcomes. ^7^ The current health challenges are not confined to a nation alone but are international, inter-sectoral, multi-level, and multi-scalar with COVID-19 being an ideal case study. ^8^^,^^9^

An approach to solving such health issues and determinants of health demands an intervention that is futuristic, context-specific, and resilient in nature and actions. Such interventions necessitate political commitment and diplomatic negotiations to reform member-driven institutions, economic reforms, cultural reforms, sensitiveness to the environment, and relevant capacity-building programs in educational institutions. The current health initiatives are issue specific with investments in addressing problems in health domains, predominantly. However, the Commission on Social Determinants of Health (CSDH) made it clear that policies for health equity involve diverse sectors with different core tasks and varied scientific traditions. ^10^ Further, the Health in All Policies (HiAP) approach to public policy systematically articulated the health impacts of decisions made outside the health sector and advocated for synergies among various sectors to avoid harmful health impacts to improve population health and health equity. It is imperative to understand policies for trade, conflict, intellectual property, economics, political science, technology, labor market, transport, supply chain, international relations etc. to develop cross-border solutions to achieve health policy outcomes. Thus, from the socioecological perspective, to address the current health policy challenges a systemic inspection is an essential requirement.

**Figure 1.**
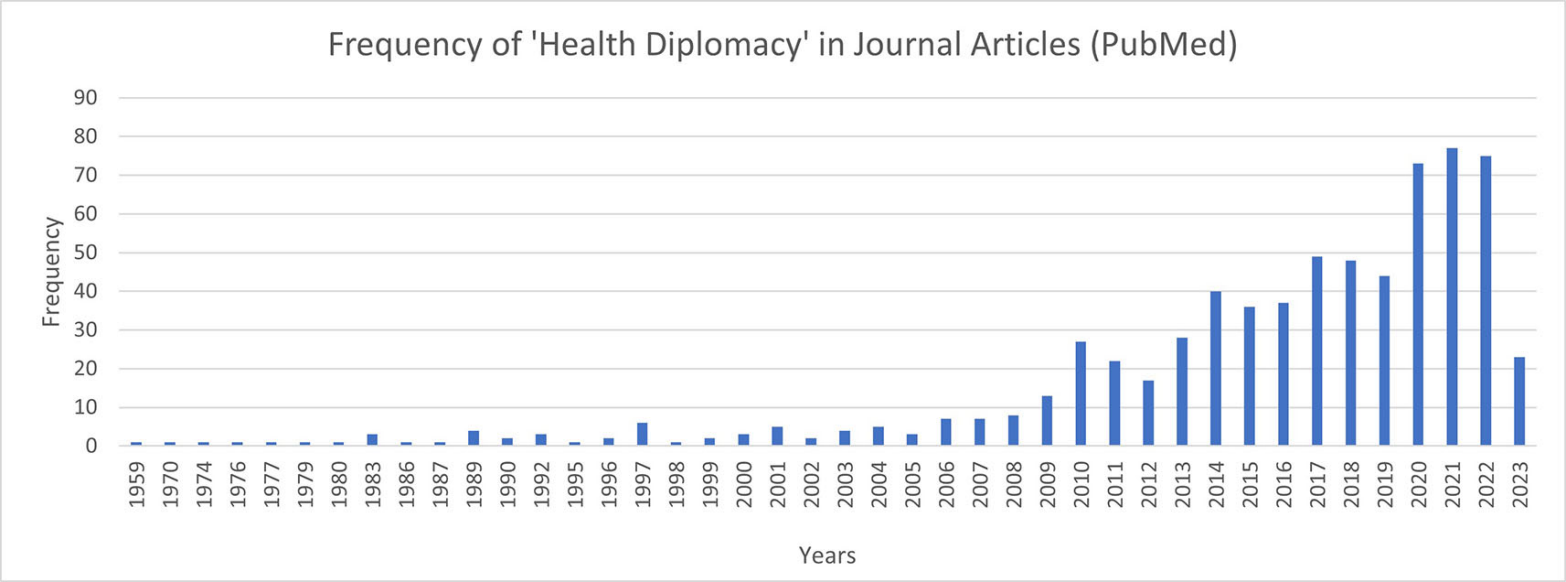
Frequency of 'Health Diplomacy' Journal Articles.

With this backdrop, one question that stands tall is to ask if there are robust educational programs covering the aforementioned issues from a health lens. Whether programs in health diplomacy cover some of the cross-cutting issues? The importance and the relevance of concept of “Global Health Diplomacy” has been increasingly recognized and practiced by both the developed and developing countries. Figure 1 depicts the frequency of health diplomacy journal articles in the last few decades in PubMed database. The graph indicates a growing interest in the academia in recent years. However, a major criticism of global health diplomacy as concept is that the deliberations are exclusionary to low- and middle-income countries that cannot compete with the negotiating power of high-income countries. ^11^ With power and influence, global health governance can potentially skew the framing of the global health agenda and marginalize issues relevant to LMICs. The increasing role of India, Indonesia and South-East Asia in global governance and health diplomacy in particular is relevant given the size of the population, disease burden and its strategic geographical location in the Indo-Pacific.

However, growing interest and influence in global health governance needs to be supported and accompanied by the necessary capacity in health diplomacy and governance. LMIC should invest in initiatives to strengthen its diplomacy skills to enhance its involvement in the staging of global health diplomacy. In this context, in this paper, a narrative synthesis of existing health diplomacy programs across the globe was conducted.

## Methods

This narrative review article reviewed the data that was extracted using an extensive web and internet search from websites of universities, massive open online courses (MOOCS), and training centres, published in English. To search the articles, search terms mentioned in Table 1 were used. Using this search method, a list of 50 programmes were assessed for eligibility. A detailed Data Extraction Sheet (DES) is presented in the Extended data. ^12^ Programmes that adopted a holistic and multidisciplinary approach to combine the fields of foreign affairs with health were included in the study. Similarly, dual degree and joint degrees programmes that aim to bring in the international relations element in public health, policy or law and vice versa were considered. The graphs and figures were derived using Microsoft Excel.

**Table 1.** List of key terms and combinations.

• Global health	• Global health and security courses
• Health Diplomacy course	• Public Health and international relations
• Public Health	• Health Diplomacy and specialisations degrees
• International relations health course	• Public health Policy and International Development
• Health diplomacy programmes	• Public Health and International relations Joint degrees
• Health Policy	• Dual Degrees and Health Diplomacy
• Public health Policy	• Health Diplomacy and Certifications
• Health Policy Concentrations	• International Affairs and Health Policy
• Global health security courses India	• Online courses and Global Health Diplomacy
• Public Policy	• Health Security and Global Health and International relations
• Public Policy and Development	• Global Health Diplomacy and Health Law
• Global Health Policy	• Community Health and International Aid and Diplomacy

Further, the programmes that were identified during the search have been divided into two broad categories:

1. **Core global health diplomacy programmes:** This group of programs includes the ones that explicitly focuses on ‘global health diplomacy’ and is mentioned in its title.
2. **Inclusive health diplomacy programmes:** This group comprises programmes that offer global health diplomacy related modules, certification, specialisation in Masters, joint or dual degrees but do not specifically mention the field in its title. The programmes identified were of various categories which the candidates would receive at its successful completion.

## Results

In the following section, the major findings have been discussed and analysed for: (i) The country where the programme/course is offered (ii) the type of global health diplomacy programme being offered and their duration (iii) Mode of teaching and (iv) The cross-cutting themes that the programme offers.

### Geographical distribution of health diplomacy programmes

**Figure 2.**
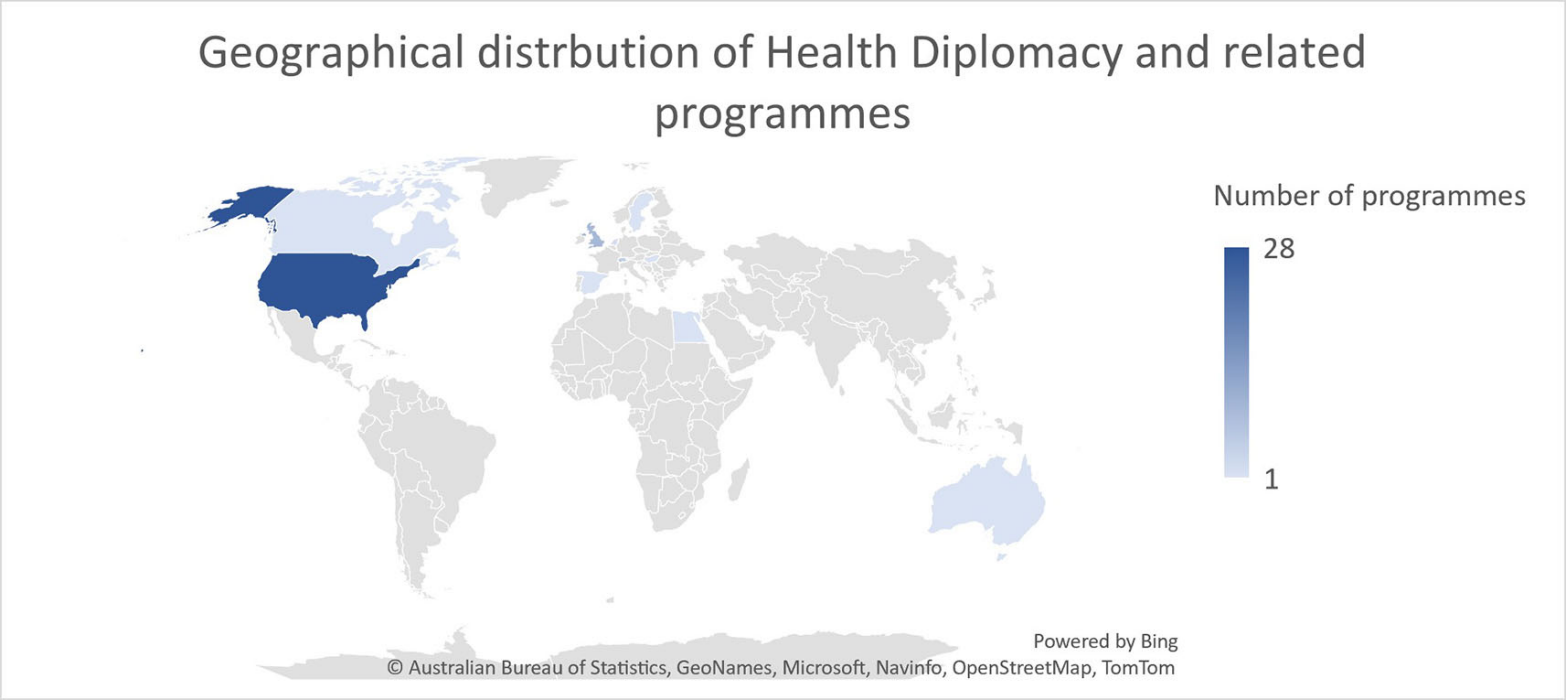
Geographical distribution of Health Diplomacy Programmes.

An overview of the geographical distribution of the global health diplomacy and the related programmes offered is depicted in Figure 2. We observe that most of the programmes/courses that deal with health diplomacy are offered in global north. The exception of the Egypt is due to the training certification offered by WHO Regional Committee for the Eastern Mediterranean Region. The highest concentration of 28 programmes and courses are in the United States of America, followed by nine in United Kingdom, five in Switzerland, and at most one offered by other countries. This state of education programmes in health diplomacy also highlights the issue of disproportionate involvement of global north in practicing health diplomacy while the global south faces greater health threats. Thus, it is imperative for the global south to train the diplomats according to the local challenges and solutions available and have an equal footing during negotiations of health-related issues at a global level.

### Types of global health diplomacy programme

**Figure 3.**
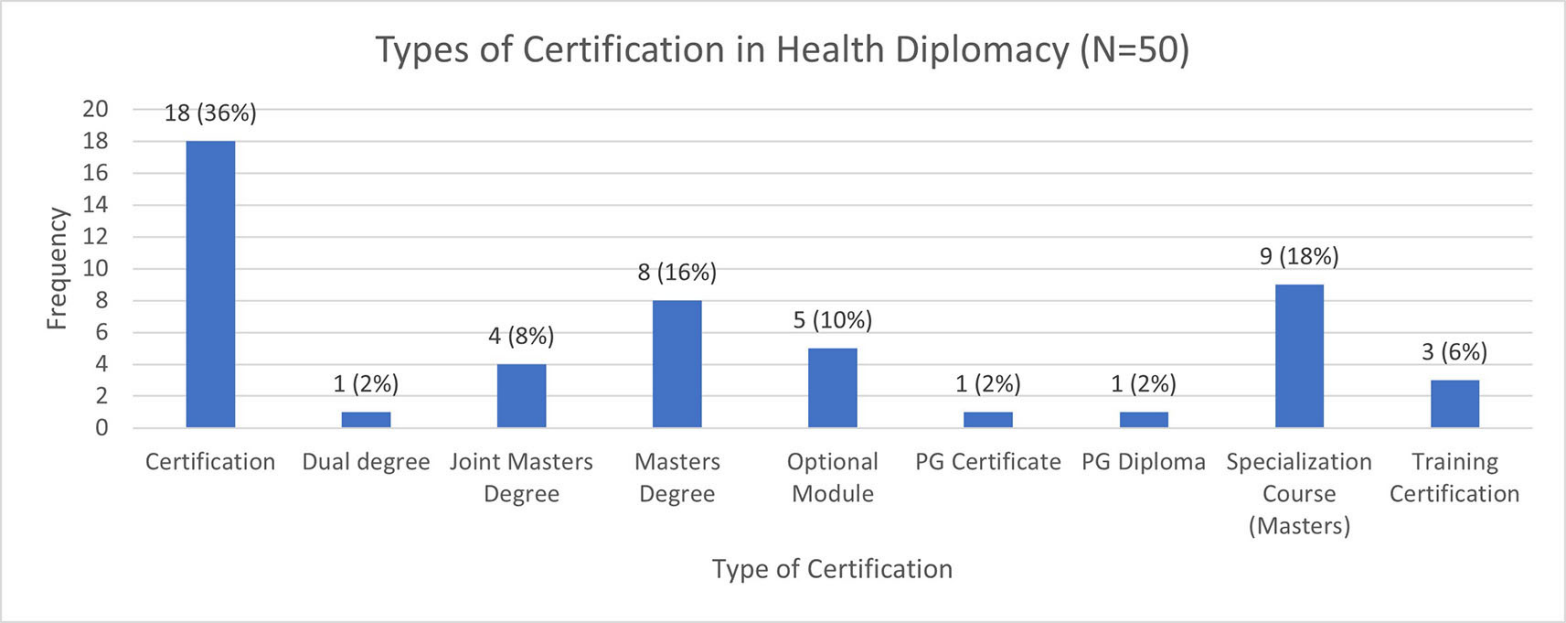
Types of Certifications in Health Diplomacy.

Overall, health diplomacy has been offered through various certification. Figure 3 depicts the type of certifications that are offer in the health diplomacy and related programmes and Table 2 provides the description of the classifications. It was seen that most of the programmes in health diplomacy and related courses were offered in the form of certificate courses. It was interesting to observe that out of the 50 programmes that were collected and examined, there were only seven programmes that could be included in the core health diplomacy programmes. This condition is interesting to ponder upon as despite the increase in the discussions of health diplomacy in academia as depicted in Figure 1, the progress in creating educational modules and training specifically for the field has been marginal. Out of the seven identified, five were certificate programmes, one was training certification, and one was in the form of an optional module. The duration allocated by these programmes was interesting to note as it had a range of highest being two months (training certification) and the lowest being 12 hours (optional module). Thus, there is need to revise the time dedicated to training of health diplomats as the relevance of this topic is in the rise more so after the COVID-19 pandemic.

**Table 2.** Classification of Type of programs.

Type of programs	Description
Master’s Degree	The category includes the programmes which offer a post graduate degree that includes Health Diplomacy and related topics. Both MSc and MA programmes have been included under this group.
Specialisation Course/Concentration in Master’s	The category includes programmes that offer a “specialisation” in the field of global health and its diplomacy that students pursue during their master’s in international relations, or public health, or law etc.
Joint Degree	Programmes of this category offers an option of attaining a degree in the field of public health with international relations, international law, or public policy jointly by two or more institutions.
Dual Degree	The category includes the programmes that involves two institutions providing master's degree in international relations in addition to a master's in public health. An enrolled candidate receives two degrees at its successful completion.
Certificate Course	This category mainly refers to non-degree granting and collaborative courses that an individual can opt for in to enhance their competencies understanding global health diplomacy.
Training Certification	This category includes programmes that were designed for practicing professionals and career diplomats among others. It is designed like a workshop where the individuals are granted certificates after the specified hours/days of teaching and training in the field of Global Health Diplomacy.
Optional Module	This category mainly comprises of open electives and modules which are offered to various master’s students as an option to choose from a list of courses within their chosen programmes. It is different from a specialisation as this covers the topic of health diplomacy marginally and are generally limited to a single semester.
PG Diploma	These programmes are less intensive than a master’s degree offered in global health policy and its diplomacy.
PG Certificate	The category of less intensive than PG Diploma with a duration of one year.

As the mentioned in the previous section, the programmes were mainly classified into two categories, and the following sub-sections provides the description of the observations that emerged during analysis.

*Core global health diplomacy programmes*

These courses were designed and delivered by USA, Canada, UK, Switzerland, Hungary, and Egypt. The curriculum in health diplomacy predominantly covered history, relevance, relevance of global institutions such as WHO, World Trade Organization (WTO), International Monetary Fund (IMF), concepts of diplomacy and governance, cross-cutting issues in relation to health in foreign policy, global health security, health system strengthening, determinants of health such as inequity, influence of trade, climate change, economics, human rights, and recent developments related to diverse health policy level challenges. Some of the competencies that are stressed were mostly cross cutting such as communication, negotiation, decision making, scenario planning, and analytical skills. Additionally, analysis of case studies and negotiation processes at the national, regional, and global levels were also covered as part of the curriculum. Table 3 provides the details regarding the seven core health diplomacy programmes offered across the world.

**Table 3.** Details of core health diplomacy programmes.

Institution	Country	Domain	Certification	Mode of training	Duration	Fee (in US Dollars) *
State University of New York	USA	Global Health Diplomacy, Geopolitics, and Finance	Certificate for Global Health Diplomacy	Online	30 hours	$29
University of Oxford	UK	International Cooperation, Global Economy, Trade and development, global health security, and strengthening health systems	Certificate for Global Health Diplomacy and Security Course	In Person	5 days	$1497
University of Toronto (Dalla Lana school of Public Health)	Canada	Global Health Diplomacy and negotiations	Certificate for Executive Course on Global Health Diplomacy	Hybrid	2 Months	$972
British Academy of Training and Development	UK	Global health diplomacy, governance, foreign policy, trade, climate change, and human rights	Certificate for Global Health Diplomacy Course	In Person	3 weeks	-
University of Pecs	Hungary	Sustainable Development goals and Global Health	Optional Module in Global Health Diplomacy	In Person	12 hours	-
Diplo	Switzerland	Global Health, foreign policy, trade, climate change, and human rights	Certificate for Global Health Diplomacy Online course	Online	9 weeks	$1042+ $274 for 4 ECTS
WHO	Egypt	Global Health Diplomacy and negotiations	Training Certification for Global Health Diplomacy	Online	2 Months	-

* The fees in terms of USD was done according to the conversion rate on May 2, 2023.

### Inclusive health diplomacy programmes

In this category, pedagogy on health diplomacy was covered briefly as part of global health, international development, international affairs, global health security, global health policy, global health affairs, international governance, public health, public policy, Humanitarian Emergencies, Global Health Law and Health System Strengthening related programs. The curriculum in health diplomacy predominantly covered was related to mostly on global health with few topics related to global health governance, political analysis, geopolitics, conflict, regulatory challenges, diplomacy, and negotiation with different stakeholders. In few programs, biological weapons, new and re-emerging diseases, demographic and epidemiological transitions, and sustainable development were discussed. However, few programs stressed the importance of protection and promotion of population health in a globalising world, at both national and transnational level. In particular, the George Washington University (Elliott School of International Affairs), covered IHR, global pandemic disease; environmental health problems; international nutrition and malnutrition; disparities in access to health care; and global health regulations and economics. A course from London School of Economics and Political Science highlights the politics of global health at the time of pandemics and learning lessons from COVID-19. The impact of conflict, climate change and migration on health was also considered in the curriculum. The course stressed on critical discussion of health-focused targets and international indicators such as the Sustainable Development Goals, the pharmaceutical industry, multilateral and bilateral donors and universal health coverage policies. And the course from Harvard University (John F. Kennedy School of Government) using the combination of lectures, interactive discussions, case studies, and group work focussed on global health security, emerging health threats, its consequences in the context of weak health systems, identifying and analysing the connections between health systems, global security policies, and international responses during conflicts.

### Mode of health diplomacy training

**Figure 4.**
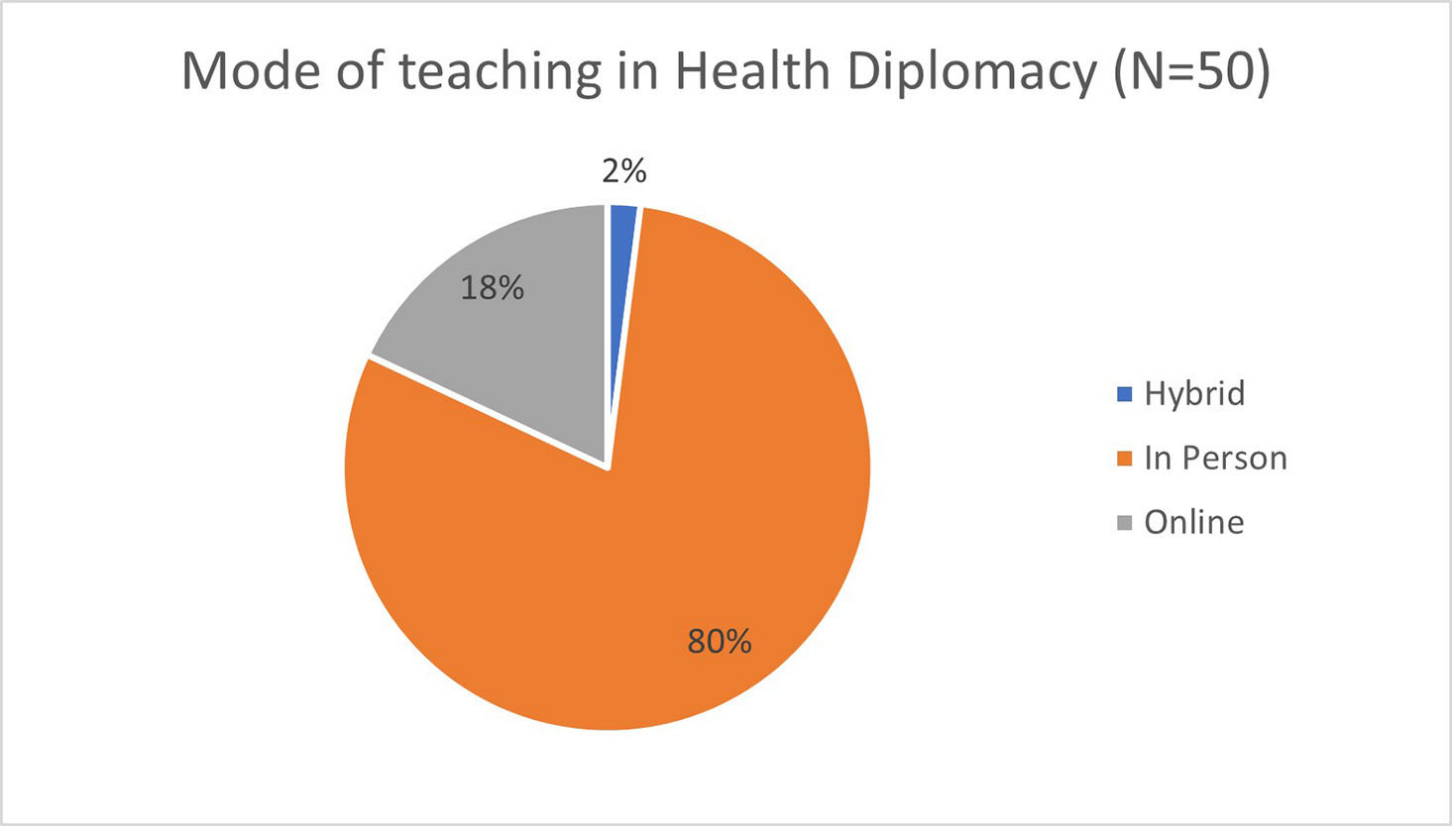
Mode of teaching in health diplomacy.

Despite the incremental increase in the use of online mode of education during the pandemic, 80 per cent of health diplomacy and the related programmes were conducted in person as shown in Figure 4. Among the seven core health diplomacy programmes, the mode of teaching used were distributed by three being online, three in-person training, and one hybrid mode. As dialogue is a key component in practicing diplomacy, it the trainers and the learners may prefer the programme to be conducted in person than in an online mode.

### Themes of health diplomacy

**Figure 5.**
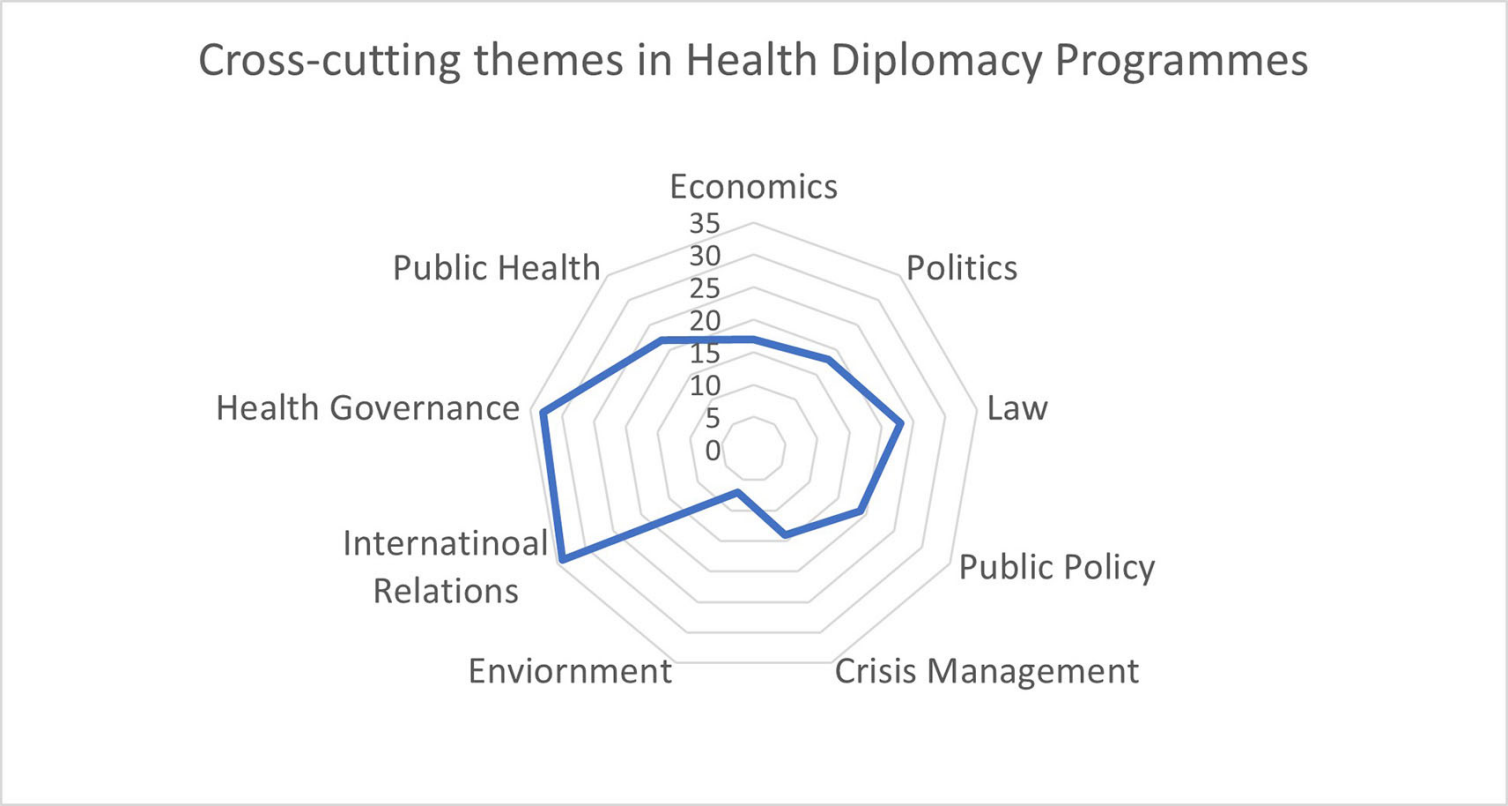
Cross-cutting themes in health diplomacy programmes.

The main component while examining in the programmes were the themes that have been included for being trained for a health diplomat by the existing programmes. Figure 5 depicts the radar diagram of the key themes that were identified in the learning outcome section of the programme/course description. We see that most of the educational content on health diplomacy has identified that health is a cross-cutting theme across various discipline such as international relations, health governance, law, politics, economics, crisis management, public policy, public health, and environment. We can observe that most health diplomacy programmes consider international relations and health governance are essential components that need to be included in the curriculum. Understanding the national and international regulations and laws were also considered to be an important for at least 23 programmes out of the 50 that were analysed.

## Discussion and conclusion

The interconnected nature of global pandemics with socioeconomic, political, geopolitical, and environmental factors was recognized as a threat to the stability of nations. A collective commitment towards minimizing the impact of a potential global pandemic was emphasized by the Oslo Ministerial Declaration in 2007 where with both foreign and health policymakers came together and stated, “We have agreed to make an impact on health a point of departure and a defining lens that each of our countries would use to examine key elements of foreign policy and development strategies”. ^13^ Foreign policy and global health were acknowledged to be interdependent for effective global health outcomes by the United Nations General Assembly (UNGA) resolutions. ^14^ During the 64th session UNGA focused on pandemic preparedness, access to diagnostics, therapeutics, and human resource for health. Foreign Policy commitments to health issues is reflected in the negotiation and adoption of the International Health Regulations (2005), the WHO Framework Convention on Tobacco Control and intergovernmental negotiations on public health, innovation, and intellectual property, and currently the ratification of IHR and proposed Pandemic Treaty.

Augmented globalization, occurrence of emerging, re-emerging diseases, COVID-19, climate change, changes in trade facilitation norms, intellectual property rights and global supply chain issues has influenced and produced uncertain conditions for negotiating global health challenges through diplomatic practices which otherwise was feasible to anticipate the solution for various global challenges including health. The current global health governance challenges are transcending both health and foreign policy dimensions. Global Health challenges have become increasingly well-known in the evolving global diplomacy agenda. There is shift in approach from “Global Health Governance to Governance of Global Health” due to recent events. Historically health was considered as “low politics” and a “mere humanitarian” endeavour in foreign policy priorities. ^15^^,^^16^ However, pandemics such as SARS, pandemic influenza, MERS, Ebola, Zika, COVID-19, Monkeypox and recent Marburg virus disease are demanding for joint commitment, cooperation among health and foreign policymakers and to consider health as “High Politics” on par with the national interests of safety, security, power, and influence. ^7^ Further, the Sustainable Development Goals, adopted in 2015, presented a major milestone in unifying efforts toward global development and partnership, thereby causing significant shift in health priorities. ^17^ Having understood the drift in global challenges and political commitment, it is imperative to ask whether the existing capacity building programs for instance in global health, international relations, health policy cover diplomatic, financial, and geopolitical context that underlies global health decision-making? And whether existing programs have a component that builds competency to describe and analyze the opportunities, challenges, and limits of Global Health Diplomacy? How many programs in health diplomacy cover competencies related to international cooperation and global solidarity, global economy, trade and development, global health security, strengthening health systems and addressing inequities to achieve global health targets? Given the policy significance of health diplomacy, authors have tried to address, create demand and build a narrative on the need for health diplomacy programs using a narrative evidence synthesis approach. The narrative synthesis has captured existing evidence on capacity building programs in health diplomacy.

Global Health Diplomacy has so far been principally defined by global institutions from developed countries. The current Global Health Diplomacy programs lack inclusivity and competency to tackle the challenges that are faced by developing countries such as access to diagnostics, vaccines and therapeutics. From the impact of COVID-19 on developing countries it was clear that health issues are transnational, and the reputed institutions in the global south should invest and put sincere effort to initiate capacity building programs in global health diplomacy in cooperation with national and international government and reputed international universities and global institutions. ^18^

The pedagogy should focus on inter-disciplinary topics such as trade, intellectual property, innovation, public policy, conflict, and human rights. The innovative methods of teaching such as scenario planning, simulation exercises and negotiation techniques would be helpful. With newer development of digital infrastructure and the associated risk of cyber security, it becomes all the more important for the Global Health Diplomacy programs to design cross-cutting curriculum that can facilitate in building transferrable skills and competencies among diverse professionals. Finally, it is a moral obligation of government, institutions, and universities both in developed and developing countries to bridge the knowledge gap in global health diplomacy. This approach can enable leaders in global health to tackle both developing and developed country problems irrespective of location using the competencies acquired in Global Health Diplomacy programs.

### Recommendation

The Figure 5 depicts the binary approach of the current health diplomacy programmes and courses. While multi-disciplinary has been vocalized in health diplomacy trainings, the potential approach to inculcate it has not been mapped in the current literature. Studies such as Bond (2008), Katz et al. (2011), Cooper & Farooq (2015), and Kickbusch et al. (2021), have highlighted the importance of including the security, trade, social justice, and development issues during the health diplomacy training. ^19^^–^^22^ Additionally, Brown et al. (2014) provided a description of the levels and the actors involved in conducting health diplomacy. ^23^ An amalgamation of the previously proposed models and the outcomes of the current study, Table 4 is a proposed framework to streamline the sectors and the competencies that is required in health diplomats.

**Table 4.** Framework of health diplomacy and competencies.

	Health Diplomacy as a security Issue	Health Diplomacy as a trade Issue	Health Diplomacy as a social justice problem	Health Diplomacy as a development issue
**Goals**	Minimize effects of conflict induced health problems and health induced conflicts (international spread of diseases)	Maximize the outcomes from the negotiations in sharing of data, technology, intellectual property, health goods and services	Address the determinants of inequity in the health systems across countries and demographics	Integrate the development goals and strategies to health outcomes such as environmental issues, energy, food, water crises
**Actors** ^23^	Attaches and Diplomats	Attaches Diplomats, Multilateral Institutional representatives, and Government agencies	Government Agency and Multilateral Institutional representatives, Country officials, Non-governmental Organizations, Universities, Public and Private Enterprises	Country officials, Non-governmental Organizations, Universities, Public and Private Enterprises
**Types of skills and courses required**	Geopolitics and international relations, conflict and health, geoeconomics, international law and health regulations, health in global context, global health crisis management, global governing institutions, negotiations skills, critical thinking, and decision making	Geoeconomics and trade, political economy, geopolitics, health in global context, geopolitics and international relations, global governing institutions, negotiation skills, critical thinking, and decision making	International law and health regulations, globalization and global inequalities, health economics, geopolitics and international relations, negotiation skills, critical thinking, and decision making	Health and SDGs, health economics, developmental studies, international law health regulations, global governing institutions, negotiation skills, critical thinking, and decision making

## Data Availability

**Open Science Framework:** Mapping Capacity Building Programs in Health Diplomacy – Relevance and Application in Uncertain World, https://doi.org/10.17605/OSF.IO/TECPS. ^12^ This project contains the following underlying data:
‐ Appendix 1: Data Extraction Sheet (DES) Data are available under the terms of the Creative Commons Zero “No rights reserved” data waiver (CC0 1.0 Public domain dedication).
