# Supplementary material for: Mapping capacity building programs in health diplomacy: Relevance and application in an uncertain world
Source: F1000Res. 2023 Jul 13;12:820. [Version 1] doi: 10.12688/f1000research.134689.1 (PMC10620483; doi:10.12688/f1000research.134689.1)
Supplement: Peer review history

# Reviewer report: Reviewer response for version 1

**DOI:** 10.5256/f1000research.147764.r188153

**Published:** 2023-11-02

**Authors:** Garry Aslanyan

**Affiliations:** 1 University of Toronto, Toronto, Ontario, Canada

The article is a good attempt to bring together current programs and course in the ever emerging area of global health diplomacy.

 There are some limitations to this review which will affect the conclusions. These include, lack of information how many of the programs/courses cited actually include simulation practical negotiation modules which are critical for diplomacy skill development?

 How many of the course participants are from global north and how many are from global south? Also, there are diplomacy courses, in various countries, that include global health as one of the subjects only - these were not included.

Is the work clearly and accurately presented and does it cite the current literature?

Yes

If applicable, is the statistical analysis and its interpretation appropriate?

Not applicable

Are all the source data underlying the results available to ensure full reproducibility?

Partly

Is the study design appropriate and is the work technically sound?

Yes

Are the conclusions drawn adequately supported by the results?

Partly

Are sufficient details of methods and analysis provided to allow replication by others?

Partly

Reviewer Expertise:

Global health, global health diplomacy

I confirm that I have read this submission and believe that I have an appropriate level of expertise to confirm that it is of an acceptable scientific standard.

# Reviewer report: Reviewer response for version 1

**DOI:** 10.5256/f1000research.147764.r188131

**Published:** 2023-11-01

**Authors:** Rajib Dasgupta

**Affiliations:** 1 Department of Community Health, Jawaharlal Nehru University, New Delhi, Delhi, India

This manuscript is an excellent review of 50 programmes/courses currently offered around the globe and four principal dimensions have been explored: i) geographical distribution; (ii)  type of global health diplomacy programme; (iii) mode of teaching and; (iv) cross-cutting themes. The study makes an important observation that these are almost exclusively limited to the global north and that cross cutting themes such as trade, law or environment need further strengthening. This article is recommended as an important contribution and a valuable addition to the discourse.

Is the work clearly and accurately presented and does it cite the current literature?

Yes

If applicable, is the statistical analysis and its interpretation appropriate?

Yes

Are all the source data underlying the results available to ensure full reproducibility?

Yes

Is the study design appropriate and is the work technically sound?

Yes

Are the conclusions drawn adequately supported by the results?

Yes

Are sufficient details of methods and analysis provided to allow replication by others?

Yes

Reviewer Expertise:

Public health policy, evalution and national health programmes

I confirm that I have read this submission and believe that I have an appropriate level of expertise to confirm that it is of an acceptable scientific standard.

# Reviewer report: Reviewer response for version 1

**DOI:** 10.5256/f1000research.147764.r188144

**Published:** 2023-11-01

**Authors:** Shyam Sundar Budhathoki

**Affiliations:** 1 Department of Primary Care and Public Health, School of Public Health, Imperial College London, London, UK

The paper is well-written account of an important area of Global Health.

 Some minor comments:

- The title mentions ‘uncertain world’. I am not sure how that is reflected in the main text. Perhaps a more neutral word/phrase could be an alternative to describe the world?
- While it is clear what the article has intended to do after reading the full paper, I feel the aims as mentioned in the main text could be a bit more elaborative. Currently it just says that ‘this paper is a narrative synthesis of existing health diplomacy programs…’. ‘Narrative Synthesis’ could be unfolded for use in aims.

Is the work clearly and accurately presented and does it cite the current literature?

Yes

If applicable, is the statistical analysis and its interpretation appropriate?

Not applicable

Are all the source data underlying the results available to ensure full reproducibility?

Yes

Is the study design appropriate and is the work technically sound?

Yes

Are the conclusions drawn adequately supported by the results?

Yes

Are sufficient details of methods and analysis provided to allow replication by others?

Yes

Reviewer Expertise:

Global Health practice and education

I confirm that I have read this submission and believe that I have an appropriate level of expertise to confirm that it is of an acceptable scientific standard.
